# Positive Effects of Elevated Platforms and Straw Bales on the Welfare of Fast-Growing Broiler Chickens Reared at Two Different Stocking Densities

**DOI:** 10.3390/ani12050542

**Published:** 2022-02-22

**Authors:** Frédérique Mocz, Virginie Michel, Mathilde Janvrot, Jean-Philippe Moysan, Alassane Keita, Anja B. Riber, Maryse Guinebretière

**Affiliations:** 1 Epidemiology, Health and Welfare Unit, French Agency for Food, Environmental and Occupational Health & Safety (ANSES), 22440 Ploufragan, France; mathildejanvrot@yahoo.fr (M.J.); jean-philippe.moysan@anses.fr (J.-P.M.); alassane.keita@anses.fr (A.K.); maryse.guinebretiere@anses.fr (M.G.); 2 Direction of Strategy and Programs, French Agency for Food, Environmental and Occupational Health & Safety (ANSES), 94701 Maisons-Alfort, France; virginie.michel@anses.fr; 3 Department of Animal Science, Aarhus University, DK-8830 Tjele, Denmark; anja.riber@anis.au.dk

**Keywords:** broiler, enrichment, footpad dermatitis, hock burn, litter quality, stocking density, walking ability

## Abstract

**Simple Summary:**

Fast-growing broiler chickens commonly experience welfare issues, such as foot and hock lesions or walking difficulties due to their genetics or the barren environment. This study assessed the impacts of elevated platforms and straw bales on the welfare of fast-growing broilers reared at two different stocking densities. The higher stocking density had negative impacts on foot and hock lesions and walking ability, whereas these welfare issues were partly positively affected by enrichments at both stocking densities.

**Abstract:**

In conventional rearing systems, fast-growing broiler chickens commonly experience welfare issues, such as contact dermatitis, walking difficulties or a lack of expression of species-specific behaviours. Enriching their environment may be a way to improve their welfare. The objective of this study was to evaluate the benefits of elevated platforms and straw bales on the welfare of fast-growing broiler chickens reared at two different stocking densities. A total of 14,994 Ross 308 broilers were housed in 12 pens according to 4 treatments: 31 kg/m^2^ with or without enrichments and 41 kg/m^2^ with or without enrichments. The broilers’ walking ability, footpad dermatitis (FPD), hock burns (HB), weight, mortality and litter quality were assessed. Stocking density had a negative effect on FPD and HB, whereas enrichments reduced the occurrence of FPD and HB at both densities. There was a positive enrichment effect and a negative density effect on body weight at 25 days and on walking ability, but no effect on the litter quality or mortality rate. These results confirm that an enriched environment improves animal welfare in confined chickens, regardless of the stocking density. Reducing stocking density clearly appears to be an important means of increasing animal welfare.

## 1. Introduction

Rearing fast-growing broiler chickens in conventional systems is commonly associated with welfare issues, such as lameness, footpad dermatitis or a lack of expression of species-specific behaviour [1]. The impairment of welfare is generally linked to fast-growing genetics and to different elements of housing systems and management, such as a high stocking density, poor litter quality or the general barren environment. Enriching the environment could improve rearing conditions and broiler welfare. According to Newberry [2], environmental enrichment is a modification of the environment of captive animals that increases the animal’s behavioural possibilities and improves biological function. There are several kinds of enrichment that can be used for broilers, e.g., elevated resting places (such as perches or platforms), panels, barriers and materials to stimulate foraging, explorative and comfort behaviours [3]. The effects of elevated platforms or perches on broiler behaviour and welfare have been the subject of recent studies that compared platforms and perches [4] or different types of platforms and configurations (number, surface, height, materials) [5,6,7] at different stocking densities [8] and studied their use on commercial farms [9] or under experimental conditions (with a small number of birds) [10,11,12]. Elevated platforms seem to be more suitable than perches for fast-growing broiler chickens due to the broilers’ weight, leg weakness and difficulties in finding their balance on “traditional” perches like bars [3]. These studies assessed several parameters, such as economics [13], health [14] and animal welfare [8,15,16,17]. The outcomes of these studies are sometimes contradictory [3], but for the studies where only a limited improvement was found in animal welfare, it may be explained by an insufficient platform surface area [5], late provision (after 7 days old) [5] or lack of access ramps [5,6].

Similarly, straw bales may be used as elevated resting places, with the additional benefit of providing the broilers with an opportunity to express normal foraging behaviour [18]. Broilers also use them to lie against when resting [19]. Riber and colleagues [3] reviewed research on the effects of these enrichments and concluded that existing studies show either no or contradictory effects on slaughter weight, mortality and locomotion. Baxter and colleagues [20] showed no effect of adding straw bales on litter quality and ammonia levels in commercially reared fast-growing chickens and found mixed results on behaviour (decrease in locomotion and increase in sitting behaviours). For Kells and colleagues, [21] straw bales had a positive effect on resting/activity, locomotion and preening behaviours in commercial farming. Bailie and O’Connell [22] studied the difference in behaviour according to two quantities of straw bales distributed (one bale per 44 m^2^ or 29 m^2^) among Ross and Cobb chickens at 30 kg/m^2^ but did not observe any differences in behaviour or leg health. Thus, the effect of straw bales on broiler behaviour and welfare appears to vary between studies.

The present study was designed to increase knowledge on the impact of environmental enrichment on the welfare of fast-growing broilers, especially in relation to leg health and walking ability. To this end, straw bales and elevated platforms with ramps to facilitate access for fast-growing broilers were provided in the rearing environment from the first day that the day-old chicks were placed there. Stocking densities usually varied between the reviewed studies and only one compared the impacts of enrichment (barrier perches with small groups of animals) according to stocking density [8]. The aim of this experiment was, therefore, to compare two different stocking densities to analyse the influence of space allowance in an enriched environment on the welfare of Ross 308 broilers reared in large groups: (a) at 41 kg/m^2^ or 31 kg/m^2^ and (b) with or without enrichment, i.e., elevated platforms with access ramps and straw bales.

## 2. Materials and Methods

### 2.1. Housing and Experimental Design

The study was conducted with 14,994 Ross 308 broiler chickens reared until 33 days of age in 6 identical rooms, each having 2 separate floor pens. The experimental design consisted of 2 × 2 modalities with 3 repetitions (3 pens) per treatment: stocking density at slaughter age of 41 kg/m^2^ or 31 kg/m^2^, with or without enrichments. 

All the pens covered 72 m^2^ (6 × 12 m) but the usable areas were considered to be 70 m^2^ in non-enriched pens, and 66 m^2^ in enriched pens as space under the feeders (i.e., 2 m^2^) and platforms (4 m^2^) were not considered as usable all the time. Indeed, we hypothesised that birds could not access the surface under the platforms during the last part of the rearing stage due to an increase in body size (the platforms being placed 30 cm off the ground). Secondly, no litter was spread on the platform surfaces, so they were not counted as usable space (in accordance with European regulations [23]). In contrast, the surface on the top of the straw bales was counted as usable since birds could access it and the straw can be considered as litter. Only the usable area was included in the calculation of the number of chicks to be placed in each pen (Table 1) in order to reach the final stocking densities (31 or 41 kg/m^2^) at 33 days of age. The different treatments in the six rooms were distributed to fit with another project on the impact of enrichments on air quality.

The bedding material used in all the pens was 1 kg/m^2^ of wood shavings with the addition of clean litter and the removal of dirty litter when necessary to maintain an acceptable condition. During the rearing period, dirty litter was removed from the most soiled areas of the pens (mainly under the drinkers) and clean litter was added 4 times in each pen from day 12. Each pen contained 3 lines of 29 nipple drinkers and 16 circular feeders. Artificial light was provided in addition to natural light, which birds had access to from 7 a.m. to 8 p.m. During the first week of age, chicks were exposed to a lighting programme of 23L:1D. From one week of age, artificial light was on from 5 a.m. to 11 p.m. The level of artificial lighting was managed by lux sensors (Tuffigo Rapidex^®^, Tuffigo Rapidex, Saint-Evarzec, France) per room depending on the level of natural light so as to ensure around 100 lux on the placement day and 30 lux from 6 days on.

### 2.2. Enrichments

An elevated perforated platform equipped with two access ramps was placed in the middle of each enriched pen. The platform, made of plastic slatted flooring, was 30 cm high, 2 m long and 1 m wide. The access ramps on either side of the platform had a 16° slope and measured 1 m × 1 m. The total surface area of the platform plus ramps was therefore 4 m^2^. We considered that 3 m^2^ was potentially accessible underneath, at least during the first week of age. These platforms were available for broilers in the enriched pens from their first to their last day of life.

One straw bale was placed on each side of the barn. These straw bales were available for broilers in the enriched pens from their first day of life. The two bales were 80 cm long, 40 cm wide and 19 cm high (2 × 0.32 m^2^ per pen). They weighed around 10 kg and were removed from their plastic packaging beforehand and tied up to ensure they stayed in position. They were not renewed if they disintegrated during the rearing period.

### 2.3. Measurements

#### 2.3.1. Litter Quality

Litter was sampled five times throughout the rearing period (once a week) in order to assess the humidity level. A handful of litter (around 10 cm diameter on the ground) was collected from four areas (between feeders) in every pen. For each pen, the samples of bedding from the four areas were manually mixed to ensure a representative sample. A subsample of approximately 20 cL was then weighed, dried for 24 h at 70 °C and reweighed to measure the dry matter [24].

#### 2.3.2. Walking Ability

Walking ability was assessed at 26 and 32 days of age on 20 randomly chosen birds in each pen. The observer walked towards one bird at a time. Birds either moved of their own volition or were stimulated vocally or by a gentle touch with the foot or hand to encourage them to walk. Scores were assigned using a 0–3 scale adapted from Meyer et al. [25] where 0 = ability to walk with no signs of lameness, 1 = unevenness in steps or stopped and sat down but able to walk 1.5 m, 2 = severe disability, birds can walk a few steps but not 1.5 m and 3 = birds unable to walk.

#### 2.3.3. Body Weight, Mortality and Contact Dermatitis

Every day during the rearing period, the number of birds that had to be culled or were found dead was recorded.

At 25 days of age, 50 sexed birds per pen (25 males and 25 females) were randomly selected for weighing (Signum 3 from Minebea^®^, Minebea Intec, Hamburg, Germany) and an evaluation of footpad dermatitis and hock burns. Contact dermatitis was assessed quite early, at 25 days, because we started to observe a high prevalence of lesions on the birds’ feet during regular inspections. To assess footpad dermatitis/hock burns, both the feet and hocks were inspected, and the worst was scored. When feet/hocks were dirty, they were gently brushed with a toothbrush and soapy water. The scoring systems were adapted from the Welfare Quality Protocol^®^ [26]: a = no evidence of footpad dermatitis/hock burns, b = minimal evidence of footpad dermatitis/hock burns (mild lesions), c = evidence of footpad dermatitis/hock burn (severe lesions). The distinction between mild and severe lesions depended on the size and depth of the lesions, according to a photographic reference [26].

#### 2.3.4. Welfare Indicators Obtained Post-Mortem

At the slaughterhouse, footpad dermatitis was evaluated on the whole batch for each treatment with an automatic camera system (Meyn^®^ footpad inspection system, Meyn, Oostzaan, Amsterdam) providing three scores, depending on the size and colour of lesions: no lesions (score a), medium/minor lesions (score b) and severe footpad dermatitis (score c). Due to the incorrect positioning of feet and other errors, only 75–95% of the pads in each batch were examined. In addition, for each treatment, carcasses were visually observed for 15 min on the slaughter line after bleeding to score hock burns with the same scoring system as used at 25 days (a = no evidence of hock burn, b = minimal evidence of hock burn, c = evidence of hock burn). A total of 1850 carcasses, i.e., hocks (both hocks were inspected, and the worst was scored), were observed per treatment, corresponding to 42–63% of the total carcasses per treatment (speed of the line: 7400 chickens per hour).

### 2.4. Statistical Analysis

The results were analysed using R (version 4.0.3) [27] and RStudio. For each of the five ages, litter humidity values were analysed using an ANOVA, with the main effects being enrichment and stocking density as well as the interaction between the two. Body weights (at 25 days and from automatic weighing scales) were analysed with the geeglm function. Pen repetition was taken into account in the analysis of manual weighing data, as was time repetition for the automatic weighing scale data. The daily cumulative mortality was analysed with a survival analysis and a Cox mixed-effects model on the number of broilers found dead during the rearing period. The walking ability scores were analysed with a generalised linear model (GLM) distinguishing birds free of lameness (score 0) from all others. To go further in the analysis, a pairwise comparison was made using the estimated marginal means model. The footpad dermatitis scores assessed on the farm were analysed with two GLMs: one distinguished score c from scores a + b to evaluate the severity of footpad dermatitis, and the other distinguished score a from scores b + c to evaluate the prevalence of lesions, whatever their severity. The hock burn scores assessed on the farm were analysed with a GLM that distinguished score a from score b (there being no or very few c scores observed). As only one data point was available per treatment (pens were not distinguished at slaughter), footpad dermatitis and hock burn at the slaughterhouse were analysed with a chi-square test for each severity score between treatments.

## 3. Results

### 3.1. Litter Quality

In the lower density, the mean levels of litter humidity varied from 25.1 ± 5.1% to 48.1 ± 7.7% in the enriched pens and from 19.4 ± 3.4% to 45.1 ± 9.9% in the unenriched pens. In the higher density, they varied from 26.8 ± 9.8% to 51.1 ± 5.2% in the enriched pens and from 22.1 ± 2.7 to 53.1 ± 8.8% in the unenriched pens. There was no effect of density (*p* = 0.55) or enrichment (*p* = 0.12) on litter humidity at any age.

### 3.2. Weight and Mortality

There was an effect of enrichment (*p* = 0.01) and of density (*p* = 0.05) on the body weight assessed at 25 days of age. Broilers from the enriched pens were heavier than those from unenriched pens, and broilers from the lower density pens were heavier than those from the higher density pens (mean body weight: 31 kg/m^2^-1376 ± 149 g with enrichment and 1357 ± 142 g without enrichment; 41 kg/m^2^-1350 ± 146 g with enrichment and 1314 ± 136 g without enrichment). No effect was found for the interaction of density and enrichment (*p* = 0.86).

The cumulative mortality rates (found dead and culled) never exceeded 5.8%. Mortality was neither affected by stocking density (*p* = 0.58), enrichment (*p* = 0.91), nor the interaction of both (*p* = 0.70).

### 3.3. Walking Ability

**Figure 1:**
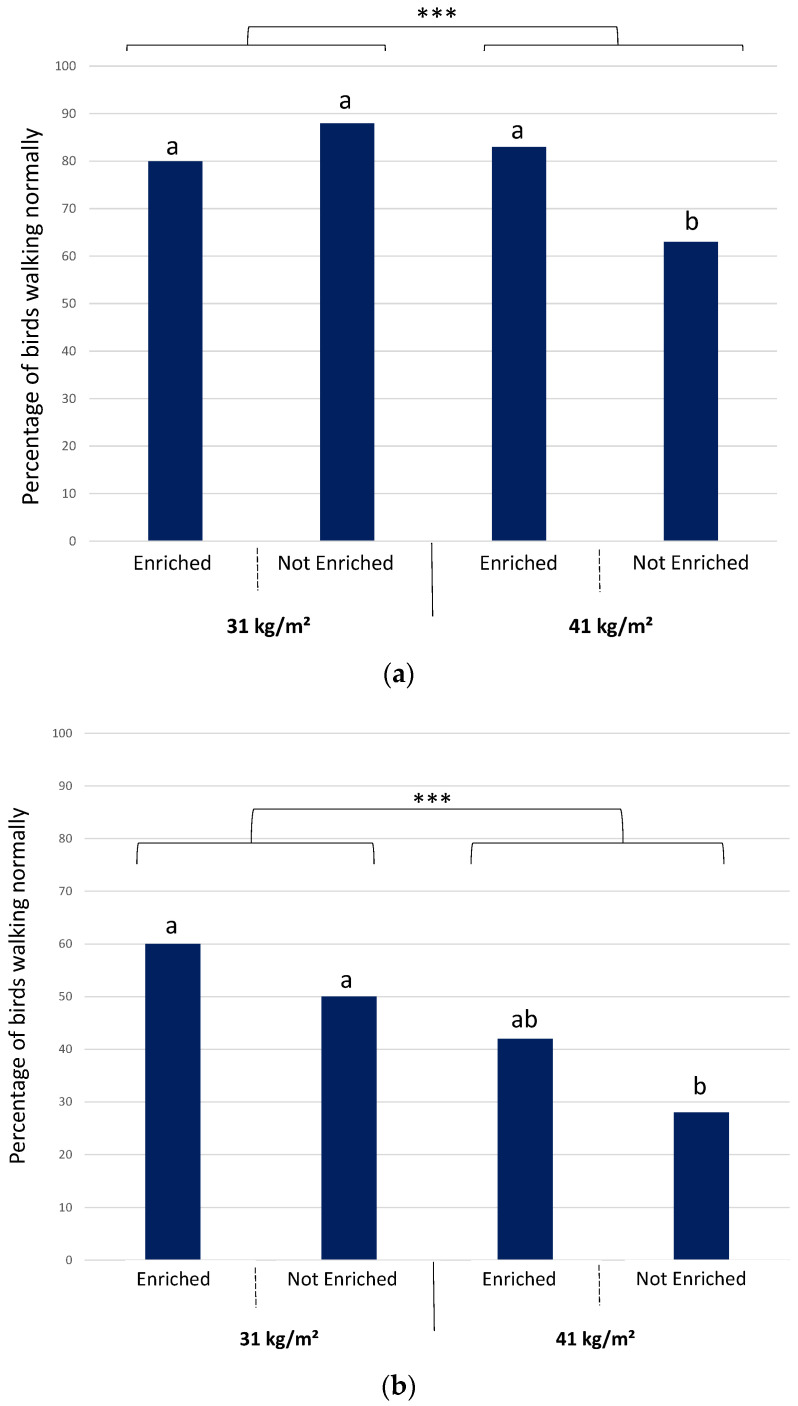
Percentage of broilers walking normally (score 0) per treatment at 26 days (**a**) and 32 days of age (**b**) (n = 60 per treatment). *** *p* ≤ 0.001. Different letters (a or b) above the columns indicate a significant difference between the groups (*p* < 0.05).

Broilers reared at the lower stocking density of 31 kg/m^2^ were able to walk better than those from the pens with a stocking density of 41 kg/m^2^ at 26 days (*p* = 0.001) and at 32 days of age (*p* = 0.0002) (Figure 1). Pairwise comparisons showed a significant effect of density in unenriched groups (*p* < 0.0001 and 0.004 at 26 and 32 days of age, respectively), whereas differences were not significant in enriched groups (*p* = 0.98 and 0.17 at 26 and 32 days of age, respectively).

Enrichment had an effect on walking ability at 26 days of age but only in the higher density groups. In groups of broilers reared at 41 kg/m^2^, there were more birds walking normally in the enriched group (83%) than in the unenriched group (63%) (*p* = 0.03) at 26 days of age. This effect disappeared at 32 days, however, though a statistical tendency remained (*p* = 0.08). This enrichment effect was not present in the lower density groups at either 26 (*p* = 0.79) or 32 days of age (*p* = 1).

### 3.4. Welfare Indicators Assessed on the Farm

#### 3.4.1. Footpad Dermatitis (FPD) at 25 Days of Age

**Figure 2:**
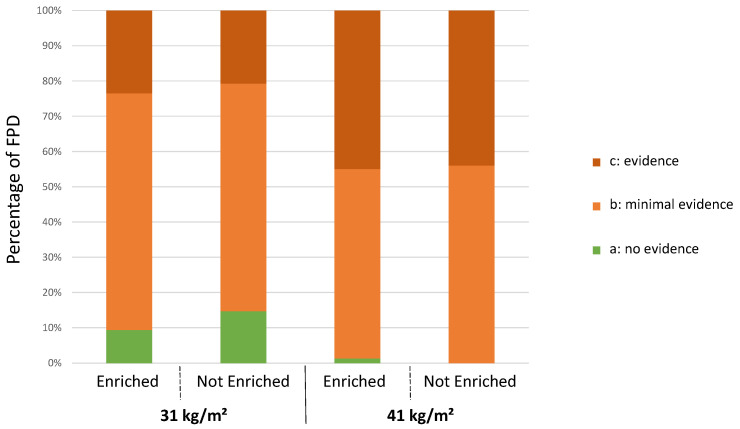
Distribution of broilers following the same treatment according to the level of footpad dermatitis (n = 150 per treatment) at 25 days of age.

An effect of stocking density on footpad dermatitis was found (Figure 2). Broilers reared at the lower density of 31 kg/m^2^ had less severe footpad dermatitis (score c) (*p* = 0.0001) and the prevalence of birds with signs of lesions (score b + c) (*p* = 0.008) was lower than those raised at the higher density of 41 kg/m^2^. There was no effect of enrichment on the percentages of severe footpad dermatitis (*p* = 0.56) or on the prevalence of lesions (*p* = 0.16).

No effect of sex was found on the level of footpad dermatitis (scores b + c: 93.5% of females and 93.8% of males) (*p* = 0.92).

#### 3.4.2. Hock Burns at 25 Days of Age

**Figure 3:**
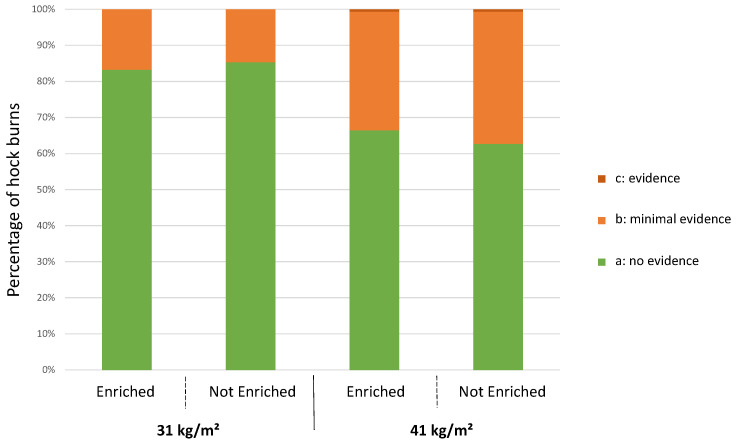
Distribution of broilers following the same treatment according to the level of hock burns (n = 150 per treatment) at 25 days of age.

Birds raised at the lower stocking density had fewer hock burns (scores b + c) than those raised at the higher density (*p* = 0.0009) (Figure 3). No impact of enrichment was found on the occurrence of hock burns (*p* = 0.62). There were so few c scores (one bird at 41 kg/m^2^ with and one bird at 41 kg/m^2^ without enrichment) that we could not compare the lesions’ severity between groups.

Males had more hock burns (scores b + c) than females at 25 days (males: 31.6%; females: 20%; *p* = 0.0009).

### 3.5. Welfare Indicators Assessed Post-Mortem

#### 3.5.1. Footpad Dermatitis

**Figure 4:**
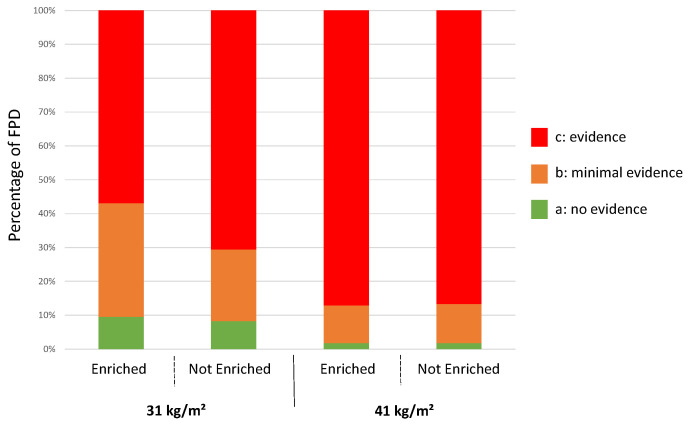
Distribution of broilers following the same treatment according to the level of footpad dermatitis observed during a post-mortem examination by an automatic camera system at the slaughterhouse.

As observed at 25 days of age, stocking density negatively impacted the levels of footpad dermatitis assessed post-mortem (Figure 4). Broiler chickens raised in pens with the higher stocking density of 41 kg/m^2^ had more severe foot lesions (score c) (*p* < 0.0001) than those at 31 kg/m^2^. There was also a lower prevalence of broilers with signs of lesions (score b + c) (*p* < 0.0001) when raised at a stocking density of 31 kg/m^2^. Minor footpad dermatitis (score b) was more common in birds raised at 31 kg/m^2^ than at 41 kg/m^2^ (*p* < 0.0001).

There was an effect of enrichments in broilers housed at 31 kg/m^2^ but not in birds reared at 41 kg/m^2^. At 31 kg/m^2^ without enrichments, more birds had severe footpad lesions (score c) (*p* < 0.0001) and fewer had minor footpad dermatitis (score b) (*p* < 0.0001) than birds raised at the same density (31 kg/m^2^) but with enrichments. There was no effect of enrichment at 31 kg/m^2^ on the absence of footpad dermatitis (score a) (*p* = 0.23). No effect of enrichment on FPD scores was observed in birds raised at 41 kg/m^2^ (score a: *p* = 1; score b: *p* = 0.58; score c: *p* = 0.43).

#### 3.5.2. Hock Burns

**Figure 5:**
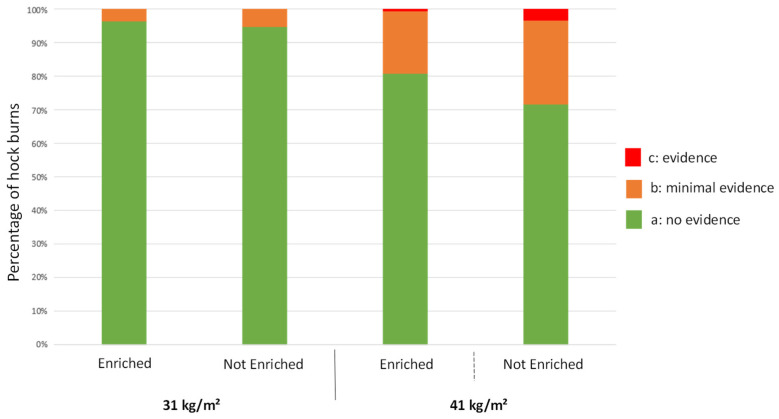
Hock burns of broilers following each treatment at post-mortem.

Stocking density and enrichment impacted the occurrence of hock burns scored on the slaughter line (Figure 5). Broiler chickens raised at the higher stocking density had more severe (score c) (*p* < 0.0001) and minor hock burns (score b) (*p* < 0.0001) than those raised at a lower density, whereas more birds raised at 31 kg/m^2^ had absolutely no sign of hock burns (score a) than those reared at 41 kg/m^2^ (*p* < 0.0001).

There was also an effect of enrichment at both 31 kg/m^2^ and 41 kg/m^2^ densities. Birds housed at 31 kg/m^2^ with enrichments had fewer minor lesions (score b) (*p* = 0.02) than those raised at the same stocking density without any enrichments. They also had no sign of hock burns (score a) (*p* = 0.02) more often than those at the same stocking density without enrichments. At 31 kg/m^2^, no severe hock burns (score c) were observed. At 41 kg/m^2^, there were more severe hock burns (score c) (*p* < 0.0001) and minor lesions (score b) (*p* < 0.0001) without enrichments than with. The opposite was observed with score a, i.e., there were more birds with no hock burns in enriched pens at 41 kg/m^2^ than in unenriched pens (*p* < 0.0001).

## 4. Discussions

The present study found that stocking density negatively impacted every measured indicator of broiler welfare except mortality, whereas enrichments had a positive effect on some of the welfare indicators, whether in one stocking density or both.

### 4.1. Contact Dermatitis and Litter Humidity

Stocking density had a negative effect on FPD and hock burns at 25 days of age. This effect was also visible in the post-mortem examination. Stocking density is often linked to welfare issues like dermatitis (e.g., [1,8]), so this result was expected. The present study shows a positive enrichment effect on FPD only in the 31 kg/m^2^ group, visible at the post-mortem examination. Nevertheless, the high level of FPD in the 41 kg/m^2^ group could have masked a potential effect of the enriched environment. Indeed, in the present study, FPD levels were high, probably due to litter management issues. Our litter was quite damp in all the pens, with moisture levels between 41.7% and 53.1% during the last week, despite the regular additions of litter. Other studies that analysed litter humidity in enriched environments measured a maximum humidity of 33% [5,12,20] (though with different experimental designs). Nevertheless, no effect of enrichment or stocking density was found on litter humidity, so the differences in FPD and hock burns between the groups could not be explained by this humidity. In the majority of previous studies, enrichments (e.g., straw bales, perches, elevated platforms and dustbathing areas) were found not to impact FPD levels [5,6,8,12,20,22,28,29]. However, in most of these studies (e.g., [6,12,29]), the great majority of birds had no signs or very low levels of FPD (i.e., between 3.88% to 7.89% of broilers observed had FPD in the study by Baxter et al. [6]). Two previous studies showed an effect of enrichment on FPD [16,18]. In the first one, Tahamtani et al. [16] demonstrated a positive effect of platforms on FPD in comparison with straw bales (groups having access to platforms 30 cm or 5 cm off the ground had better FPD scores than groups having access to straw bales only). In the second study, Ohara et al. [18] also found a positive effect of enrichments, i.e., straw bales and perches, but only on the foot lesions of females. This difference in FPD between males and females was not observed in the present study but the assessment was conducted earlier (25 days) and on another strain (Ross 308) than in Ohara’s study [18] (Japanese broilers, assessment of FPD at 60 days old, at slaughter). However, we found more hock burns in males at 25 days old. The cause of hock burns is multifactorial, i.e., hock burns may be related to inactivity [30], growth rate [31], litter moisture [32], genetics [33] or body weight [1,34,35]. The difference between the levels of hock burns in males and females could be explained by the body weight, males being heavier than females. Beyond the sex effect, a positive enrichment impact was noted on hock burns observed at slaughter. In contrast to FPD, where the enrichment only affected the broilers from the lower density of 31 kg/m^2^, there was a positive enrichment effect on hock burns at both densities, with broilers from the enriched groups having fewer hock burns than those from the unenriched groups. To the authors’ knowledge, no previous study has shown an effect of enrichment (i.e., barrier perches, elevated platforms, ramps, straw bales, dustbathing area) on levels of hock burns in broiler chickens (with [8] or without comparing stocking densities [5,6,22,28,29]). Thus, the present study shows that enrichment, such as elevated platforms and straw bales, may improve hock health as well as footpad health. Future research could be carried out to see whether elevated platforms and straw bales have the same positive impact on leg health or whether differences are also observed between straw bales and perches [18].

### 4.2. Walking Ability

Our finding that stocking density negatively impacts walking ability at 26 and 32 days of age is consistent with the literature showing evidence of a decrease in walking ability when density is increased (e.g., [1]). However, in pairwise comparisons, this negative effect of stocking density was found only in the groups of broilers without access to enrichments. Thus, the presence of enrichments seems to mitigate the negative consequences of stocking density on walking ability. As there was no general significant effect of enrichments, this result nonetheless needs to be further examined with more repetitions and more or different enrichment materials. Generally, in previous studies on platforms and straw bales, no effect of these enrichment materials was found on walking ability [5,6,9,12,20,22]. However, in these studies, walking difficulties were rarely observed, unlike in the present study where the number of broilers with walking difficulties (scores 1, 2 and 3) was quite high, which may explain why an effect of enrichment was observed. The exception is the study by Kaukonen et al. [9], whose results are in agreement with ours in that they show a positive effect of elevated platforms on the mean gait score of broilers. Finally, it is possible that the poor litter quality observed in our study gave us the opportunity to demonstrate both enrichment and density impacts on contact dermatitis and walking ability, whereas other studies rarely observed such welfare issues.

### 4.3. Mortality and Weight

Our results showing no effect of either enrichment or stocking density on mortality are consistent with the literature. In previous studies on the same genotype (Ross 308) with different types of enrichment (straw bales, various shapes and the height of perches and platforms), whether on commercial farms or in experimental facilities, enrichments did not impact mortality rates [5,6,7,20]. No effect of stocking density on mortality is commonly found in the literature (e.g., [8,36,37]).

In contrast, body weights were slightly impacted by both enrichment and stocking density at 25 days of age. Broilers reared at a density of 31 kg/m^2^ were heavier than those from the higher density of 41 kg/m^2^**.** This result is consistent with previous studies showing a negative impact of stocking density on body weight (e.g., [36,37]). Furthermore, broilers from enriched pens were heavier than those from unenriched ones at 25 days. We can hypothesise that increased activity due to the use of enrichments leads to more muscle mass and to heavier birds. To compare this finding with the literature, the effect of enrichment is in agreement with Ohara et al. [18], who found a greater final body weight among Tatsuno slow-growing broilers in enriched pens (straw bales and perches) than among controls. In contrast, De Jong et al. [29] found that broilers (males from two strains: Ross 308 slaughtered at 38 days and JA757 slaughtered at 53 days) reared without any enrichment were heavier from day 17 onwards than birds reared with enrichments (barrier perches, ramps, platforms and a dustbathing area). De Jong et al. [29] concluded that enrichments increased the activity of birds, which then had an adverse effect on performance (average body weight and other parameters). This conclusion differs from that of Ohara et al. [18], who also observed increased activity in an enriched environment but deduced that enhancing broilers’ activity with enrichments may not have adverse effects on productivity.

### 4.4. Platform Use and Impact on Stocking Density

In the present study, the surfaces below and above the platforms were, for different reasons, not counted as usable areas in the enriched pens. However, broilers perched on the platform throughout the rearing period, at times covering the entire surface of platforms and ramps (personal observations). Moreover, the area underneath the platforms was fully occupied by the broilers throughout the rearing period, mostly for the purpose of resting. Thus, the effective stocking density, if including the platform surfaces (below and above), was around 27–28 kg/m^2^ as compared to the 31 kg/m^2^ in lower density pens and 37–38 kg/m^2^ as compared to the 41 kg/m^2^ in higher density pens. Thus, the positive enrichments effects observed (on weights, walking ability, FPD, and hock burns) cannot be completely differentiated from the lower stocking density impact. The addition of enrichments in the rearing environment can then be considered as positive, intrinsically due to the increased possibilities for the expression of natural behaviours like perching and foraging, but also due to the increase in space allowance that it comes with.

## 5. Conclusions

Our results suggest that providing elevated platforms and straw bales helps to improve broiler welfare by reducing footpad dermatitis, hock burns and walking difficulties even at a high stocking density. However, reducing stocking density remains the key to improving broiler welfare. Further investigations are needed to deepen the knowledge of the effect of enrichments on birds’ walking ability and to distinguish between the effects of different types of enrichments, examined separately, using a variety of stocking densities.

## Figures and Tables

**Table 1:** Distribution of broilers per treatment in the 12 pens of the 6 rooms.

Density	41 kg/m^2^						31 kg/m^2^					
Room	Room 1		Room 2		Room 3		Room 4		Room 5		Room 6	
Enrichment	Yes		No		Yes	No	Yes	No	No		Yes	
Pen	1	2	3	4	5	6	7	8	9	10	11	12
Number of broilers	1385	1385	1471	1471	1385	1471	1040	1103	1103	1103	1040	1040

## Data Availability

Data is contained within the article.
